# In Vivo Analysis of Tissue S-Nitrosothiols in Pediatric Sepsis

**DOI:** 10.3390/antiox13030263

**Published:** 2024-02-21

**Authors:** Daniel T. Cater, Charles Clem, Nadzeya Marozkina, Benjamin Gaston

**Affiliations:** 1 Division of Critical Care, Department of Pediatrics, Riley Hospital for Children, Indiana University School of Medicine, Indianapolis, IN 46202, USA; dancater@iu.edu; 2 Division of Pulmonology, Department of Pediatrics, Riley Hospital for Children, Indiana University School of Medicine, Indianapolis, IN 46202, USA; cclem@iu.edu (C.C.);; 3 The Herman B. Wells Center for Pediatric Research, Indianapolis, IN 46202, USA; 4 Crossroads Pediatric Device Consortium, Indianapolis, IN 46202, USA

**Keywords:** S-nitrosothiols, pediatrics, sepsis, pediatric intensive care unit, equipment design

## Abstract

S-nitrosothiols are endogenous, bioactive molecules. S-nitrosothiols are implicated in many diseases, including sepsis. It is currently cumbersome to measure S-nitrosothiols clinically. We have previously developed an instrument to measure tissue S-nitrosothiols non-invasively using ultraviolet light. We have performed a prospective case control study of controls and children with sepsis admitted to the PICU. We hypothesized that tissue S-nitrosothiols would be higher in septic patients than controls. Controls were patients with no cardiopulmonary instability. Cases were patients with septic shock. We measured S-nitrosothiols, both at diagnosis and after resolution of shock. A total of 44 patients were enrolled: 21 controls and 23 with sepsis. At baseline, the controls were younger [median age 5 years (IQR 0, 9) versus 11 years (IQR: 6, 16), *p*-value = 0.012], had fewer comorbidities [7 (33.3%) vs. 20 (87.0%), *p*-value < 0.001], and had lower PELOD scores [0 (IQR: 0, 0) vs. 12 (IQR: 11, 21), *p*-value < 0.001]. S-nitrosothiol levels were higher in sepsis cohort (1.1 ppb vs. 0.8 ppb, *p* = 0.004). Five patients with sepsis had longitudinal measures and had a downtrend after resolution of shock (1.3 ppb vs. 0.9 ppb, *p* = 0.04). We dichotomized patients based on S-nitrosothiol levels and found an association with worse clinical outcomes, but further work will be needed to validate these findings.

## 1. Introduction

Sepsis is a leading cause of morbidity and mortality worldwide [1]. Globally, there are an estimated 8 million deaths from sepsis [2]. Sepsis is one of the leading causes of death in the pediatric population; over one-third of global deaths from sepsis occur in children [3]. Vascular dysfunction is common in sepsis and is a large driver of both mortality and development of multiple organ dysfunction syndrome [4,5]. Nitrosative stress has been implicated in the pathophysiology of sepsis [6], and increased nitrosative stress has been associated with both severity of sepsis and organ dysfunction [7]. Despite this, various therapies targeting nitrosative stress pathways have failed to improve outcomes in sepsis [8,9,10]. Nitric oxide’s role in sepsis is complex and has both favorable and unfavorable actions. A more complete understanding of nitrosative stress in sepsis is necessary to better tailor therapies.

S-nitrosothiols are a class of endogenous molecules that represent important effectors of nitrogen oxide bioactivities. These compounds contain a nitroso group attached to sulfur. S-nitrosothiols are involved in numerous physiologic pathways including blood pressure regulation, regulation of respiratory drive, and airway smooth muscle tone [11,12,13,14,15]. Low-mass S-nitrosothiols are important signaling molecules that are predominantly produced by metalloproteins such as ceruloplasmin [16], hemoglobin [17], and NO synthase [13]. Importantly, these low-mass S-nitrosothiols have cGMP independent bioactivities [13,18,19]. Previously, S-nitrosothiol levels have been measured ex vivo and found to be elevated in adult patients with sepsis [17]. While these compounds have been implicated in many disease processes including sepsis, they are labile; thus, measurement is difficult and currently limits its clinical utility. Recently, our team has designed and validated a novel approach to measure S-nitrosothiols in vivo [20]. This device uses ultraviolet light homolytically to break the S-nitrosothiol bond, producing NO. NO can diffuse through the skin and be measured using a NO analyzer. Our previous work has demonstrated both the sensitivity and specificity of this device: it measures nM quantities of endogenous S-nitrosothiols, such as S-nitrosocysteine and S-nitrosoglutathione, and does NOT detect either nitrite or nitrate at physiological pH. This technology detects S-nitrosothiols in rats in vivo. Our objective here was to characterize tissue S-nitrosothiol levels in vivo in pediatric patients with sepsis. We hypothesized that pediatric patients with sepsis would have higher levels of S-nitrosothiols as measured by our device. We also hypothesized that higher S-nitrosothiol levels would be associated with worse clinical outcomes.

## 2. Materials and Methods

We performed a prospective study of pediatric patients admitted to the pediatric intensive care unit from 2021–2023. Patients with sepsis were eligible for enrollment if they were admitted to the intensive care unit with a diagnosis of sepsis and were on vasoactive support. Controls were primarily enrolled in the PICU, and were patients without requiring any vasoactive support (an additional four patients without hemodynamic compromise were included from the pulmonary clinic). Patients were excluded if they had a chronic skin condition or burn excluding measurement of S-nitrosothiol levels, or if they were on inhaled NO therapy. This study was performed in accordance with Indiana University (IRB#10839). Informed consent was obtained from participant’s legal guardian prior to study enrollment. Assent was also obtained for patients aged 8–17 years unless their clinical situation precluded capacity for providing assent.

For S-nitrosothiol measurements, we used a device our team previously described [20]. In brief, we used a UV light device (SV003 10W Alonefire, Shenzhen, China) which produces light at a 365 nm wavelength, as the UV source, and a 12 mm diameter double convex focusing lens with a 24 mm effective focal length. A custom 3D printed housing for the light, focusing lens, and side port tube were used. This device was then connected by a side port and Teflon tubing to an EcoPhysics NOA (Ann Arbor, MI, USA) to detect NO. A full measurement included a 10 s ambient air recording with the UV light off, a 10 s measurement with the device on the skin surface but with the light off, and a 10 s measurement with the device on the skin and the light on. Measurements were obtained in triplicate for each patient. Measurements were obtained at the palmar wrist, shoulder, and ear. To obtain final values reported, the ambient measurement was subtracted from the measurement obtained with device on the skin and with the UV light on. Significant exhaled breath contamination was noted for measurements obtained at shoulder and ear, and therefore only wrist measurements were reported.

Patient demographics, laboratory data, PICU interventions, length of stay data, and medication data were collected from the electronic medical record. Pediatric Logistic Organ Dysfunction (PELOD) scores at the time of PICU admission were extracted from Virtual Pediatric Systems (VPS, LLC http://www.myvps.org/, accessed on 15 August 2023) database. Vasoactive inotrope scores [21,22] were calculated at multiple time points. Development of acute respiratory distress syndrome (ARDS) was conducted using the Pediatric Acute Lung Injury Consensus Conference (PALICC-2) guidelines [23]. Development of multiple organ dysfunction syndrome (MODS) was calculated using Goldstein’s criteria [24]. We calculated both MODS and ARDS development in the first 24 and 72 h after measurement of S-nitrosothiol levels. Exploratory outcomes included composite 28-day measures of the number of ventilator-free days, days free from vasopressor use, days alive and out of the ICU, and days alive and out of hospital.

Data were summarized and distributions were examined. Categorical variables were presented as counts and frequencies and were compared using chi-squared or Fisher’s exact as appropriate. Non-Gaussian continuous variables were presented as medians with interquartile ranges and compared using the Wilcoxon rank sum test. Paired-sample Wilcoxon tests were performed for paired measurements. Multiple linear regressions were performed to compare associations between S-nitrosothiol levels and relevant clinical outcomes. To attempt to control for cohort type, regression models with cohort type as a covariate were constructed. Logistic regression models were fitted to evaluate the performance of photolysis measurements in distinguishing septic patients from controls. An optimal cut-point was determined for S-nitrosothiol levels measured by our device using Liu’s index [25]. Receiver operator characteristic (ROC) curves were generated and the area under the ROC curve was denoted after fitting logistic regression models. A two-tailed *p*-value of <0.05 was considered statistically significant. Data were analyzed using STATA 17 [26].

## 3. Results

A total of 44 patients were enrolled with a median age of 7.5 years (IQR: 2.5, 15). There were 23 cases enrolled and 21 controls. There were three (6.8%) non-survivors in the cohort. The controls were younger [median age 5 years (IQR 0, 9) vs. 11 years (IQR: 6, 16), *p*-value = 0.012]. However, there was no relationship between age and S-nitrosothiol measurement r = 0.28, *p* = 0.060. The controls also had fewer comorbidities [7 (33.3%) vs. 20 (87.0%), *p*-value < 0.001] and lower PELOD scores [0 (IQR: 0, 0) vs. 12 (IQR: 11, 21), *p*-value < 0.001]. There were no differences in gender, ethnicity, race, or BMI (see Table 1).

### 3.1. S-Nitrosothiol Levels Stratified by Case or Control

**Figure 1:**
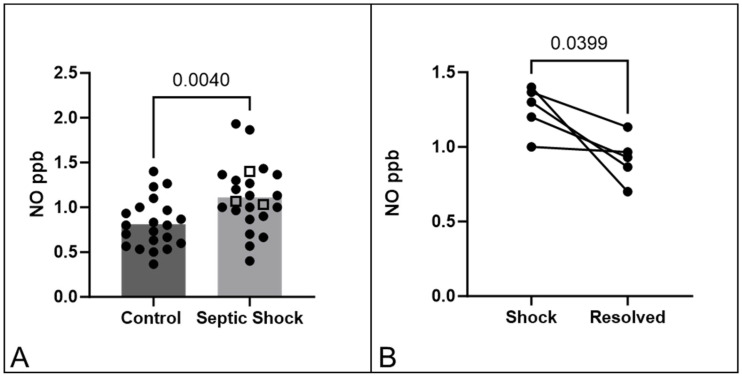
(**A**) Photolytic measurement readings obtained at the wrist. Left (dark grey) bar depicts control patients. Right (light grey) bar depicts patients with septic shock. The open square boxes denote the three patients who died. *Y*-axis is nitric oxide (NO) measured by device in parts per billion. Patients with septic shock had significantly higher levels of NO measured by device when compared to controls (*p*-value = 0.004). (**B**) Graphical representation of photolytic measurements obtained during shock (left) and after resolution of shock (right). Photolytic measurements obtained after resolution were statistically significantly lower than the measurements obtained during shock state.

Our primary outcome was difference in S-nitrosothiol levels as measured by our device between cohorts of patients. Patients with sepsis had statistically significantly higher NO measurements compared with controls [1.07 ppb (0.9, 1.4) vs. 0.8 ppb (0.6, 0.97), *p*-value = 0.004]. Figure 1A denotes this difference. Five patients with sepsis had repeat measurements of S-nitrosothiol levels after resolution of shock. There was a significant decrease in S-nitrosothiol level after shock resolution [1.3 ± 0.16 ppb vs. 0.9 ± 0.16 ppb, *p*-value = 0.04] (see Figure 1B).

### 3.2. Determination of Abnormal Photolysis Measurement

**Figure 2:**
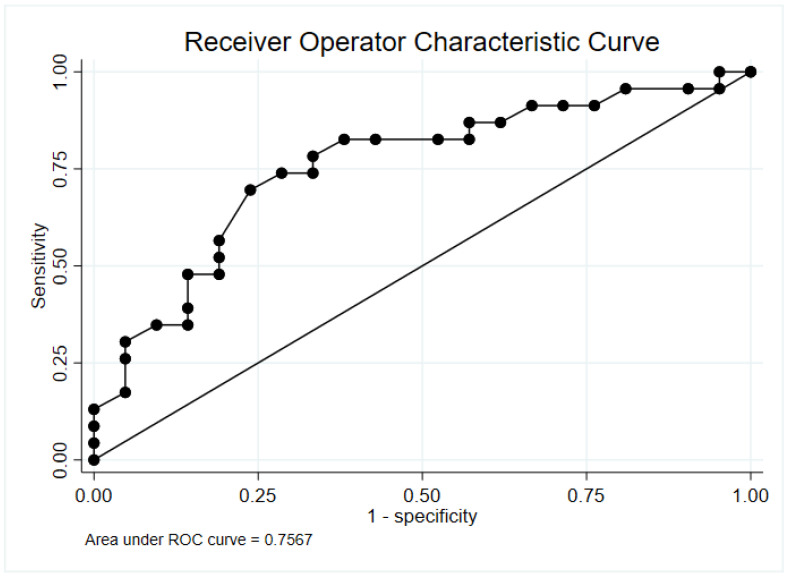
Receiver Operator Characteristic Curve for S-nitrosothiol measurement. Area under the receiver operator characteristic curves are denoted in the bottom left (AUC = 0.7567).

In our cohort, the optimal cut-point for abnormal photolytic measurement was 1.0 ppb based on Liu’s index. Figure 2 demonstrates the Receiver operator characteristic (ROC) curve. The area under the ROC curve was 0.757. A photolytic measurement greater than or equal to 1.0 ppb had a 70% sensitivity and 76% specificity for identifying a septic patient vs. a control (See Table 2).

The cohort was then dichotomized into normal and abnormal photolytic measurement based on a level of greater than or equal to 1.0 ppb. Patients with a photolytic reading greater than or equal to 1.0 ppb had higher PELOD scores [0.5 (0, 12) vs. 12 (10, 21), *p*-value = 0.008], oxygenation indices [0 (0, 0) vs. 2 (0, 10), *p*-value = 0.011], vasoactive inotrope scores [0 (0, 6) vs. 8 (2, 15), *p*-value = 0.002], and higher odds of MODS development [7 (36.8%) vs. 15 (79.0%), *p*-value = 0.020] in addition to higher amounts of dysfunctional organs [0 (0, 2) vs. 2 (0, 3), *p*-value = 0.008]. Patients with photolytic measurements greater than or equal to 1.0 ppb also had fewer ventilator-free days, vasoactive-free days, and days free from the hospital (See Table 3).

### 3.3. Association of S-Nitrosothiol Levels with Length of Stay

S-nitrosothiol levels measured by our device were associated with longer lengths of stay data. Specifically, hospital and ICU LOS, as well as length of invasive mechanical ventilation and length of vasoactive requirement, were all increased as S-nitrosothiol levels increased (see Table S1). Multivariate linear regression models were constructed for S-nitrosothiol levels and length of stay data controlling for whether participants were controls or septic patients. After controlling for the participant cohort type, higher S-nitrosothiol levels trended toward an association with longer length of hospital and ICU stays [Hospital LOS Beta coefficient: 1.4 (−0.0 to 2.7), *p*-value = 0.052; ICU LOS Beta coefficient: 1.2 (−0.2 to 2.5), *p*-value = 0.083] (see Table S2).

### 3.4. Association of S-Nitrosothiol Levels with MODS and ARDS Development

Higher S-nitrosothiol levels were associated with higher odds of both MODS and ARDS development at multiple time points (see Table S3). One ppb increase in NO measured by our device was associated with 12-fold increased odds of development of MODS in the following 24 h, and 12.9-fold increased likelihood of development of MODS in the following 72 h (*p*-values = 0.039 and 0.043, respectively). Likewise, a one ppb increase in NO measured by our device was associated with 15.3-fold increased odds of development of ARDS in the following 24 h (*p*-value = 0.030).

### 3.5. Correlations of S-Nitrosothiol Levels with Sepsis Biomarkers

Higher S-nitrosothiol levels were correlated with multiple clinical biomarkers. Higher S-nitrosothiol levels were correlated with higher vasoactive inotrope scores (Spearman’s correlation coefficient = 0.45, *p*-value = 0.002), higher PELOD scores (Spearman’s correlation coefficient = 0.44, *p*-value = 0.003), and higher number of dysfunctional organs (Spearman’s correlation coefficient = 0.48, *p*-value = 0.001). There was no correlation with S-nitrosothiol levels and serum lactate measurement at admission (Spearman’s correlation coefficient 0.21, *p*-value = 0.415) (see Figure S1).

## 4. Discussion

This study is the first study to test this novel non-invasive device in a cohort of patients with sepsis. S-nitrosothiol levels, as measured by our device, were significantly higher in patients with sepsis compared to controls. This study also identified a cut-off value to determine abnormal S-nitrosothiol levels as measured by our device. Additionally, when septic patients were dichotomized into normal and abnormal S-nitrosothiol levels, an abnormal S-nitrosothiol level was associated with worse exploratory clinical outcomes.

Similarly to what has been demonstrated ex vivo in both animal models [27,28] and humans [17,28], our in vivo models confirmed that S-nitrosothiol levels were higher in patients with sepsis compared to controls. S-nitrosothiols are produced to counter multiple infections [29,30,31], and it has been demonstrated that S-nitrosothiols likely have important roles in both the amelioration and the pathophysiology of sepsis [28]. Doctor and coworkers have previously shown higher levels of S-nitrosylated hemoglobin in patients with systemic inflammatory response syndrome and acute respiratory distress syndrome [17]. Liu et al. further validated this work, demonstrating higher levels of S-nitrosylated hemoglobin in adult patients with gram-negative sepsis [28]. Our work adds to this by demonstrating higher total S-nitrosothiol levels, as measured in the PICU in situ, in pediatric patients with sepsis.

Given that S-nitrosothiols play a complex role in sepsis, it is important to attempt to establish normal and abnormal levels of S-nitrosothiols. In this study we attempted to do this. Using Liu’s index, we were able to determine an optimal cut-point to stratify patients as having sepsis or not. For our device, we found the S-nitrosothiol reporter molecule, NO, to be 1.0 ppb NO. Further work will be needed to validate this cut-point in a larger cohort of patients. In addition, future work will need to be undertaken to validate the S-nitrosothiol level readings from our device compared to measurements of common S-nitrosothiols from the patients’ blood.

In this cohort of patients, after dichotomizing our cohort into normal and abnormal S-nitrosothiol levels, we demonstrated a number of clinically relevant outcomes that were worse in the cohort of patients with abnormal S-nitrosothiol levels. We demonstrated higher oxygenation indices in patients with abnormal S-nitrosothiol levels, which is consistent with prior human studies examining nitrosylated hemoglobin levels in patients with acute respiratory distress syndrome [17]. Similarly, we demonstrated worse hemodynamics, evidenced by a higher vasoactive inotrope score in those with abnormal S-nitrosothiol levels as measured by our device. This is also consistent with prior studies. Basal S-nitrosothiol levels have been demonstrated to increase vasodilation in bioassays [32,33], and lower blood pressure has been seen when nitrosylated hemoglobin is infused intravenously [34]. While there are plausible mechanistic reasons for worsening clinical outcomes in patients with higher S-nitrosothiol levels, this work needs to be further validated in a larger cohort of patients.

Our study has some limitations; it was a small pilot study and therefore a larger study is needed to validate the findings. This work will also need to be further replicated to establish normal and abnormal readings. Another limitation of our study is no internal measurements of S-nitrosothiol levels found in patient blood. We plan to perform this as a next step in validation of this device. Lastly, we need to determine whether the abnormal levels identified here can lead to interventions that affect outcomes.

## 5. Conclusions

Using a new non-invasive device, we were able to measure S-nitrosothiol levels in vivo for a cohort of patients with and without sepsis. Higher S-nitrosothiol levels were seen in patients with sepsis compared to controls. When dichotomized, abnormal S-nitrosothiol levels may be associated with worse clinical outcomes, but further work will be needed to validate these findings.

## Figures and Tables

**Table 1:** Baseline Demographics.

Variable	Control (n = 21)	Sepsis (n = 23)	*p*-Value
Age	5 (0, 9)	11 (6, 16)	0.012
BMI	16.8 (15.8, 19.2)	18.1 (14.5, 24.5)	0.805
Gender			0.919
Female	7 (33.3%)	8 (34.8%)	
Male	14 (66.7%)	15 (65.2%)	
Ethnicity			0.999
Hispanic	0 (0.0%)	1 (4.4%)	
Non-Hispanic	21 (100.0%)	22 (95.7%)	
Race			0.895
Asian	1 (4.8%)	0 (0.0%)	
Black	2 (9.5%)	2 (8.7%)	
Unknown	0 (0.0%)	1 (4.4%)	
White	14 (85.7%)	20 (87.0%)	
Presence of Comorbidities	7 (33.3%)	20 (87.0%)	<0.001
PELOD ^1^	0 (0, 0)	12 (11, 21)	<0.001

^1^ PELOD = Pediatric logistic organ dysfunction score.

**Table 2:** Descriptive statistics for abnormal photolytic measurement.

Statistical Measures	Photolytic Measurement ≥ 1 ppb
Sensitivity	0.70
Specificity	0.76
Area under ROC curve	0.73

**Table 3:** The 28-day outcomes and severity of illness data stratified by photolytic measurements.

Variable	Normal Photolytic Reading (<1 ppb)	Abnormal Photolytic Reading (≥1 ppb)	*p*-Value
PELOD	0.5 (0, 12)	12 (10, 21)	**0.008**
Vasoactive Inotrope Score	0 (0, 6)	8 (2, 15)	**0.002**
Oxygenation Index	0 (0, 0)	2 (0, 10)	**0.011**
ARDS Development	4 (21.1%)	8 (42.1%)	0.163
MODS Development	7 (36.8%)	15 (79.0%)	**0.020**
Number of Dysfunctional Organs	0 (0, 2)	2 (0, 3)	**0.008**
28-Day Outcomes	Beta-Coefficient	95% Confidence Interval	*p*-Value
Ventilator-free days	−5.0	−9.8 to −0.2	**0.043**
Vasoactive-free days	−4.6	−8.6 to −0.5	**0.027**
ICU-free days	−2.9	−10.0 to 4.2	0.408
Hospital-free days	−7.0	−13.4 to −0.5	**0.035**

Liu’s method was used to determine optimal cut-point for normal vs. abnormal values. PELOD = Pediatric Logistic Organ Dysfunction score; ARDS = Acute respiratory distress syndrome; MODS = Multiple organ dysfunction syndrome; ICU = Intensive care unit. The 28-day outcomes are composite outcomes of days alive and free from ventilator, hospital, ICU, or vasoactive support. Significant *p*-values are denoted by bold font.

## Data Availability

The data presented in this study are available on request from the corresponding author. The data are not publicly available due to privacy concerns.

## Supplementary Materials

The following supporting information can be downloaded at: https://www.mdpi.com/article/10.3390/antiox13030263/s1, Table S1: Log-transformed linear regressions of photolytic readings and length of stay; Table S2: Log-transformed multivariate linear regressions of photolytic reading and length of stay; Table S3: Logistic regressions of photolytic readings and both development of MODS and ARDS. Figure S1: Correlations of multiple clinical biomarkers and S-nitrosothiol levels.
