# Supplementary material for: In Vivo Analysis of Tissue S-Nitrosothiols in Pediatric Sepsis
Source: Antioxidants (Basel). 2024 Feb 21;13(3):263. doi: 10.3390/antiox13030263 (PMC10967417; doi:10.3390/antiox13030263)
Supplement: Supplementary file 1 [file antioxidants-13-00263-s001.zip › antioxidants-2809364-supplementary.pdf]

Variable	Beta-coefficient	95% Confidence Interval	p-value
**Hospital LOS**	2.5	1.05 to 3.9	**0.001**
**ICU LOS**	2.4	0.9 to 3.9	**0.002**
**Length of IMV**	2.4	0.3 to 4.6	**0.025**
**Length of vasoactive support**	2.6	0.9 to 4.3	**0.004**

Table S1. Log transformed linear regressions of photolytic readings and length of stay. LOS=length of stay; IMV=Invasive mechanical ventilation.

Variable	Beta-coefficient	95% Confidence Interval	p-value
**Hospital LOS**	1.4	-0.0 to 2.7	0.052
**ICU LOS**	1.2	-0.2 to 2.5	0.083
**Length of IMV**	-0.1	-1.5 to 1.4	0.935
**Length of vasoactive support**	0.1	-0.4 to 0.6	0.620

Table S2. Log transformed multivariate linear regressions of photolytic readings and length of stay. Model controlled for whether patient was healthy control or septic. LOS=length of stay; IMV=Invasive mechanical ventilation.

Variable	Odds Ratio	95% Confidence Interval	p-value
**MODS 24**	12.0	1.1 to 126.8	**0.039**
**MODS 72**	12.9	1.1 to 154.5	**0.043**
**ARDS 24**	15.3	1.3 to 177.7	**0.030**
**ARDS 72**	7.3	0.9 to 57.0	0.056

Table S3. Logistic Regressions of photolytic readings and both development of MODS and development of ARDS within 24 and 72 hours of photolytic measurements. MODS=multiple organ dysfunction syndrome; ARDS=acute respiratory distress syndrome.

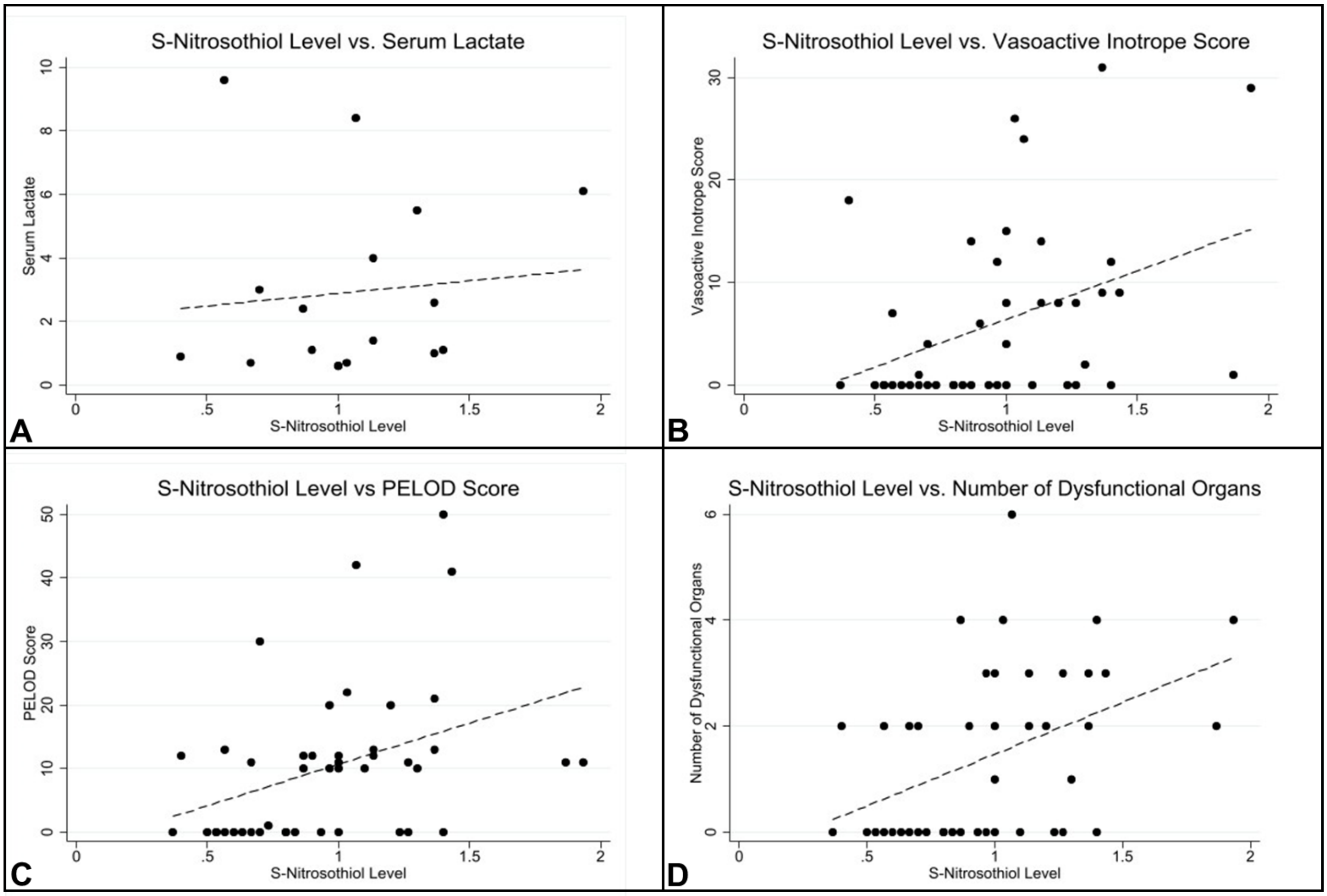

Figure S1. Correlations of multiple clinical biomarkers and S-nitrosothiol Levels. (A) Correlation of initial serum lactate level and S-nitrosothiol levels (Spearman's correlation coefficient=0.21, p-value 0.415). (B) Correlation of highest vasoactive-inotrope score and S-nitrosothiol level (Spearman's Correlation Coefficient=0.45, p-value 0.002). (C) Correlation of PELOD score and S-nitrosothiol level (Spearman's Correlation Coefficient=0.44, p-value 0.003). (D) Correlation of number of dysfunctional organs and S-nitrosothiol level (Spearman's Correlation Coefficient=0.48, p-value 0.001).
